# Management of Sinus Node Dysfunction After AF Ablation: Beyond a Conservative Strategy

**DOI:** 10.1111/anec.70201

**Published:** 2026-05-14

**Authors:** Osman Karaarslan, Mehmet Mustafa Yılmaz, Macit Kalçık

**Affiliations:** ^1^ Department of Cardiology Hitit University Erol Olçok Education and Research Hospital Corum Turkey; ^2^ Department of Cardiology, Faculty of Medicine Hitit University Corum Turkey

## Author Contributions

All of the authors contributed planning, writing, and revision.

## Funding

The authors have nothing to report.

## Conflicts of Interest

The authors declare no conflicts of interest.

To the Editor,

We read with interest the recent article by Guo et al. investigating the temporal characteristics and management of prolonged sinus pause (PSP) following atrial fibrillation termination after catheter ablation. The authors reported that most PSP episodes resolved within 1 week and suggested that a conservative watch‐and‐wait strategy may be appropriate in many patients (Guo et al. 2026). While these findings contribute valuable observational data to an underexplored clinical scenario, several methodological and interpretative aspects deserve further discussion before generalizing this management approach.

The interpretation of PSP reversibility should be considered cautiously in view of the limited sample size and the absence of systematic evaluation of baseline sinus node function prior to ablation. Sinus node dysfunction may remain clinically silent in patients with atrial fibrillation and can become apparent only after restoration of sinus rhythm, making it difficult to distinguish transient post‐procedural effects from pre‐existing intrinsic disease. Previous electrophysiological observations have demonstrated that termination of atrial fibrillation can unmask latent sinus node dysfunction independent of procedural injury (Hocini et al. 2003). Therefore, the assumption that early PSP primarily reflects reversible post‐ablation mechanisms may not fully capture the complexity of underlying sinus node physiology.

Another important consideration relates to the potential contribution of sinus node artery injury during left atrial ablation. The anatomical variability of the sinus node artery and its proximity to commonly targeted ablation regions may predispose selected patients to transient or permanent sinus node dysfunction. Experimental and clinical studies have shown that thermal injury along the course of the sinus node artery may lead to reversible bradyarrhythmias, but in some cases persistent dysfunction requiring pacing therapy may occur (Choi et al. 2013). In this context, the absence of post‐procedural anatomical imaging limits mechanistic interpretation of PSP episodes observed in the study population.

Furthermore, discontinuation of antiarrhythmic therapy immediately after detection of PSP represents another potential confounding factor in the interpretation of recovery patterns. Antiarrhythmic drugs are known to influence sinus node automaticity and atrial conduction properties, and their withdrawal may itself modify the natural history of post‐ablation bradyarrhythmias. Previous clinical observations suggest that recovery of sinus node function after catheter ablation frequently occurs within the first 1–2 weeks, but this recovery trajectory may reflect combined pharmacological and procedural effects rather than a purely transient ablation‐related phenomenon (Kitamura et al. 2016).

Finally, although the proposed watch‐and‐wait strategy appears reasonable in selected asymptomatic patients, the absence of a structured risk stratification algorithm limits its broader clinical applicability. Early identification of patients at risk for persistent sinus node dysfunction remains essential to avoid delayed pacemaker implantation in symptomatic individuals. Prior studies have demonstrated that acute sinus node dysfunction after atrial ablation is not uniformly benign and may require individualized monitoring strategies based on clinical and procedural characteristics (Killu et al. 2016). Larger prospective multicenter investigations incorporating standardized electrophysiological assessment and imaging‐guided evaluation of sinus node artery integrity may help refine decision‐making in this challenging clinical setting.

Sincerely,

Osman Karaarslan, Mehmet Mustafa Yılmaz, and Macit Kalçık

## Data Availability

Data sharing not applicable to this article as no datasets were generated or analysed during the current study.
